# SARS-CoV-2 Infection, Risk Perception, Behaviour, and Preventive Measures at Schools in Berlin, Germany, during the Early Post-Lockdown Phase: A Cross-Sectional Study

**DOI:** 10.3390/ijerph18052739

**Published:** 2021-03-08

**Authors:** Franziska Hommes, Welmoed van Loon, Marlene Thielecke, Igor Abramovich, Sascha Lieber, Ralf Hammerich, Sabine Gehrke-Beck, Elisabeth Linzbach, Angela Schuster, Katja von dem Busche, Stefanie Theuring, Maximilian Gertler, Gabriela Equihua Martinez, Joachim Richter, Clara Bergmann, Alisa Bölke, Falko Böhringer, Marcus A. Mall, Alexander Rosen, Alexander Krannich, Jan Keller, Norma Bethke, Marco Kurzmann, Tobias Kurth, Valerie Kirchberger, Joachim Seybold, Frank P. Mockenhaupt, BECOSS Study Group

**Affiliations:** 1 Institute of Tropical Medicine and International Health, Charité—Universitätsmedizin Berlin, 13353 Berlin, Germany; welmoed.van-loon@charite.de (W.v.L.); marlene.thielecke@charite.de (M.T.); elisabeth.linzbach@charite.de (E.L.); stefanie.theuring@charite.de (S.T.); maximilian.gertler@charite.de (M.G.); gabriela.equihua-martinez@charite.de (G.E.M.); joachim.richter@charite.de (J.R.); clara.bergmann@charite.de (C.B.); alisa-boelke@web.de (A.B.); frank.mockenhaupt@charite.de (F.P.M.); 2 Clinic for Anesthesiology, Charité—Universitätsmedizin Berlin, 13353 Berlin, Germany; igor.abramovich@charite.de (I.A.); sascha.lieber@charite.de (S.L.); 3 Medical Directorate, Charité—Universitätsmedizin Berlin, 13353 Berlin, Germany; norma.bethke@charite.de (N.B.); marco.kurzmann@charite.de (M.K.); kirchberger@heartbeat-med.de (V.K.); joachim.seybold@charite.de (J.S.); 4 Clinical Quality and Risk Management, Charité—Universitätsmedizin Berlin, 13353 Berlin, Germany; ralf.hammerich@charite.de; 5 Institute of General Practice, Charité—Universitätsmedizin Berlin, 13353 Berlin, Germany; sabine.gehrke-beck@charite.de (S.G.-B.); angela.schuster@charite.de (A.S.); 6 Department of Pediatric Surgery, Charité—Universitätsmedizin Berlin, 13353 Berlin, Germany; katja.von-dem-busche@charite.de; 7 Labor Berlin—Charité Vivantes Services GmbH, 13353 Berlin, Germany; falko.boehringer@laborberlin.com; 8 Department of Pediatric Pulmonology, Immunology and Critical Care Medicine, Charité—Universitätsmedizin Berlin, 13353 Berlin, Germany; marcus.mall@charite.de (M.A.M.); alexander.rosen@charite.de (A.R.); 9 Clinical Study Center, Charité—Universitätsmedizin Berlin, 13353 Berlin, Germany; alexander.krannich@charite.de; 10 Department of Education and Psychology, Freie Universität Berlin, 14195 Berlin, Germany; jan.keller@fu-berlin.de; 11 Institute of Public Health, Charité—Universitätsmedizin Berlin, 13353 Berlin, Germany; tobias.kurth@charite.de

**Keywords:** SARS-CoV-2, Coronavirus, COVID-19, school, children, seroprevalence

## Abstract

Briefly before the first peak of the COVID-19 pandemic in Berlin, Germany, schools closed in mid-March 2020. Following re-opening, schools resumed operation at a reduced level for nine weeks. During this phase, we aimed at assessing, among students and teachers, infection status, symptoms, individual behaviour, and institutional infection prevention measures. Twenty-four primary and secondary school classes, randomly selected across Berlin, were examined. Oro-nasopharyngeal swabs and capillary blood samples were collected to determine SARS-CoV-2 infection (PCR) and specific IgG (ELISA), respectively. Medical history, household characteristics, leisure activities, fear of infection, risk perception, hand hygiene, facemask wearing, and institutional preventive measures were assessed. Descriptive analysis was performed. Among 535 participants (385 students, 150 staff), one teenager was found to be infected with SARS-CoV-2 (0.2%), and seven individuals exhibited specific IgG (1.3%). Compared to pre-pandemic times, screen time (e.g., TV, gaming, social media) increased, and the majority of primary school students reported reduced physical activity (42.2%). Fear of infection and risk perception were relatively low, acceptance of adapted health behaviors was high. In this post-lockdown period of low SARS-CoV-2 incidence in Berlin, individual and school-level infection prevention measures were largely adhered to. Nevertheless, vigilance and continued preventive measures are essential to cope with future pandemic activity.

## 1. Introduction

Early in the COVID-19 pandemic, school closures were globally implemented as a central containment intervention. However, school closures bear the risk for several adverse long-term social and economic effects on children and society [1], including widening disparities [2], lowered mental health [3] and physical activity [4], and a loss of (health-care) workforce [5]. Increased screen time (e.g., use of social media, gaming, and watching television) during school closures is observed but, so far, little is known about potentially adverse outcomes [6]. The benefits and disadvantages of school closures continue to be intensely debated [7]. In Berlin, Germany, schools attended by approximately 360,000 pupils were closed from 17 March 2020 until 27 April 2020. Following gradual re-opening, teaching under strict hygiene measures with limited pupil numbers and reduced schedules continued until 25 June 2020, when the summer holidays started. During these nine weeks between school-reopening and the summer break, SARS-CoV-2 transmission in Berlin was comparatively low; 2376 cases were recorded by the local health authorities. The reported 7-day incidence in this period varied between 4.9 and 13.6 cases per 100,000 inhabitants [8]. While transmission of SARS-CoV-2 in school settings is feared, existing evidence argues against a major role for schools in driving, rather than mirroring, the pandemic. Previous data suggest that children are rarely the index cases of clusters [9,10,11]. In Germany, the Robert Koch Institute (i.e., the national public health institute) recorded 48 COVID-19 school outbreaks (≥2 cases) between January and August 2020, constituting 0.5% of all reported outbreaks in that period. These school outbreaks included 216 cases, of which only 30 occurred among children aged 6–10 years, whereas most cases were present in those 21 years of age and older. As compared to the time before school closure, re-opening coincided with a slight increase of mean outbreaks per week (3.3 vs. 2.2) and mean cases per outbreak (6 vs. 4) [12]. Another analysis concluded that school re-openings had not increased the number of newly confirmed SARS-CoV-2 infections in Germany [13]. School outbreaks are recognized based on symptomatic infections. However, upon SARS-CoV-2 infection, children tend to present with milder symptoms [14,15], and up to 50% of them may stay asymptomatic [16]. The actual number of infected children attending school might thus be higher than deduced from outbreaks. On the other hand, child-to-child transmission appears to be lower than transmission from and between adults [11,17,18]. Limited evidence suggests that in young children (e.g., below 10 years of age) susceptibility to SARS-CoV-2 infection is lower than in adults and that infectivity increases with age. These findings are less pronounced among secondary school attendees [10]. Studies from Germany show significantly lower seroprevalence rates in young children compared with adults [16].

School reopening in Germany was accompanied by official recommendations for infection prevention and control (IPC) measures, including health behaviours, such as hand hygiene, physical distancing, wearing facemasks, as well as self-isolation, and testing of symptomatic students and staff. Implementation of these measures could significantly reduce disease transmission in schools [19]. Nevertheless, their effectiveness largely depends on individual adherence, which may be influenced by behavioural beliefs such as risk perception and fear of infection [20]. A notable scarcity of data actually obtained in schools. Therefore, during the early post-lockdown and low incidence phase in June 2020, the present study aimed at investigating the SARS-CoV-2 infection situation as well as individual and institutional adaptations to the pandemic among primary and secondary school students and school staff attending 24 classes, randomly selected across Berlin. Specifically, we aimed at assessing (i) the prevalence of SARS-CoV-2 infections, seroprevalence, and prevailing symptoms; (ii) the parameters describing current attitudes, activities, and fears; (iii) individual level health behaviours; and iv) the implementation of institutional IPC measures in the school setting,. This paper presents the baseline data of our longitudinal study spanning one year.

## 2. Materials and Methods

### 2.1. Study Design, Setting, and Participants

This is a cross-sectional analysis of a longitudinal study performed in 24 Berlin school classes. The present first round of examinations was conducted between 11 and 19 June 2020, which will be followed by three further rounds at intervals of roughly three-months. For the selection of schools, the twelve districts of Berlin were divided into three socio-economic strata according to the city’s Social Atlas [21]. In a random selection process, two districts per stratum were selected and in each of these two primary and two secondary schools were chosen. Three schools refused to participate (two because of organisational concerns, one because of an expected low participation rate) and were replaced by substitutes of the same stratum. Per facility, one class was chosen by the school to account for organisational necessities. In primary schools, classes were selected amongst grades 3–5, in secondary schools they were selected amongst grades 9–11. We aimed to examine 20 students per class and up to 10 school staff with direct contact to the students per class (teachers, educators). Written study information was provided to potential participants at least one week prior to the school visit to obtain written consent from parents or legal guardians, and study staff were available for questions via telephone.

### 2.2. Data Collection

Study teams visited the schools at scheduled dates. At the visits, body temperature was measured by forehead scanners, with fever defined as a temperature of ≥37.5 degrees Celsius. A brief medical history was obtained, including fever, acute respiratory symptoms, and loss of smell or taste. Combined oropharyngeal/naso-pharyngeal swabs (eSwab, Copan, Italy) were professionally collected, and finger-prick blood samples were taken on filter paper (Bio Sample Card, Ahlstrom Munksjö, Helsinki, Finland). Consenting study participants who were absent during the school visit due to reported disease were visited at home, usually on the same day. SARS-CoV-2 infection was determined by real-time-PCR (Roche Diagnostics, Rotkreuz, Switzerland) within 24 h after swab collection. Anti-SARS-CoV-2-IgG was assessed by punching 4.75 mm discs from dried blood spots, extracting samples in a 250 µL sample buffer at ambient temperature for 1 h, and performing ELISA on a EUROLabWorkstation (Euroimmun AG, Lübeck, Germany).

A week before the study visit, participants were asked to complete a paper-based questionnaire (versions were adapted for children, adolescents, and adults) assessing, among other variables, signs and symptoms, household composition, contacts to positive cases, risk factors, fear of infection, risk perception towards SARS-CoV-2 infection, health behaviours including hand hygiene, physical distancing, and facemask wearing, and leisure activities, for the latter items comparing the present to the pre-pandemic situation. One-item assessments were used; response scales in aggregated form were depicted. Lastly, school-level IPC measures were documented to examine the grade of implementation of official recommendations [22]. For this, class teachers completed a questionnaire on implementation of these IPC measures, including (1) basic hygiene measures, e.g., hand hygiene; (2) keeping distance; (3) absence rule for symptomatic persons; (4) fresh-air ventilation, at least once/school break; (5) cohort building of learning groups; (6) changes to the subjects taught: physical education outside, no choir/theatre/orchestra rehearsals; (7) staggering of teaching hours; and (8) home-schooling for staff and students belonging to risk groups.

### 2.3. Data Processing and Statistical Analysis

All data collection was done in a pseudonymised manner on paper forms and was subsequently digitalised and managed using REDCap electronic data capture tools hosted at Charité—Universitätsmedizin Berlin [23]. Descriptive analyses comprising calculation of percentages and the median and range of age in years were segregated for primary school students, secondary school students, and school staff. We used R version 3.6.3 for all analyses.

## 3. Results

### 3.1. Participants

In 12 primary and 12 secondary schools, 535 participants were enrolled in the study, including 193 (36%) primary school students, 192 (36%) secondary school students, and 150 (28%) school staff. The inclusion rate of students (i.e., enrolled students by all students per class) was 65% (range, 13–96%). The median age (range) was 10 (8–13) years for primary school students and 15 (13–18) for secondary school students; half (190/383) were male. Staff participants comprised of 75.7% (112/148) teachers and educators and 24.3% (36/148) facilitating personnel. The majority of school staff were female (71.0%; 98/138), and the median (range) age was 46 (28–65) years. Compliance with sample collection was high: none of the staff refused to be swabbed and only 0.7% (1/150) refused the finger prick test; among students, 0.8% (3/385) refused to be swabbed and 0.8% (3/385) refused the finger prick test.

### 3.2. Prevalence of SARS-CoV-2 Infection and IgG Antibodies

We detected one SARS-CoV-2 infection among 532 participants (0.2%): a 16-year old, afebrile female student who reported headache and rhinorrhoea as the only symptoms upon enrolment. For the 14 days before enrolment, she reported a cough, headache, rhinorrhoea, and limb pain, and she was unaware of any contact with a positive case. Seven participants (1.3%; 7/527) amongst all students showed IgG antibodies to SARS-CoV-2; three of them belonged to one secondary school class. The median (range) age of the sero-reactive students was 14 (9–17) years and 5/7 were female. One of them reported a loss of smell and taste within the preceding two weeks.

### 3.3. Signs and Symptoms, and Chronic Conditions

At examination, fever was present in 2.1%, 3.1%, and 1.3% of primary school students, secondary school students, and staff, respectively (Table 1). Any sign or symptom on the examination day was reported by 19% of primary school students, 16% of secondary school students, and 12% of the staff. Headache was one of the leading symptoms among all groups, but individual symptoms differed between students and school staff. Loss of smell or taste was reported rarely and exclusively by students (0.5%). Signs and symptoms in the preceding 14 days were reported by 41%, 55%, and 48% of primary school students, secondary school students, and staff, respectively (Table 1).

Chronic diseases were more frequently reported by staff (44%) than by primary and secondary school students (13% and 18%, respectively). Among staff, high blood pressure was the most common chronic condition (14%), whereas for students this was lung disease (4% for both primary and secondary school students) (Table 1).

### 3.4. Household Characteristics and Leisure Activities

Most participants lived in households of 3–4 persons (primary school students, 60.6%, 117/193; secondary school students, 74.9%, 143/191; staff, 39.3%, 59/150). Overall, households more often contained kindergarten children (primary school students, 26.8%, 51/190; secondary school students, 8.4%, 16/191; staff, 23.7%, 27/114) rather than people above the age of 60 years (13.2%, 25/189; 9.1%, 17/186; 19.3%, 22/114, respectively). Most students had their own room at home (primary school students, 71.1%, 138/193; secondary school students, 87.4%, 167/191). Among students, contacts to a suspected COVID-19 case in the preceding 14 days were rarely reported (primary school students, 1.1%, 2/190; secondary school students, 1.6%, 3/187) as were contacts to confirmed cases (1.1%, 2/189; 0.5%, 1/189, respectively). Staff reported such contacts more often (contact to a suspected case and confirmed case in 3.5%, 5/143, and 0.7%, 1/149, respectively). The main mode of transport to school was walking for primary school students (53.2%, 101/190), public transport for secondary students (50.5%, 96/190), and car driving for staff (54.0% (81/150).

**Figure 1:**
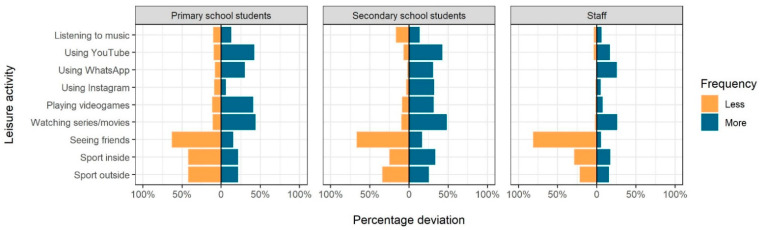
Deviations of time spent on leisure activities within the preceding 14 days compared to pre-pandemic times. Note: missing values to 100% reflect no change of activities.

Changes in leisure time activities compared to pre-pandemic times are displayed in Figure 1. Spending time with friends was greatly reduced across all three subgroups (63–81%). A clear reduction of physical activity was seen only for primary school students. In contrast, substantial proportions of students reported increases in “screen time”. For instance, more than 40% of the primary and secondary students stated spending more time with YouTube and TV than prior to the pandemic. Screen time increased particularly among children compared to adults (Figure 1).

### 3.5. Fear of Infection and Risk Perception

Fear and risk perception were pronounced among staff: about half of them reported medium to very strong fear of infection, and 59% reported moderate to very high perceived risk of infection, whereas these figures were lower among students (medium to very strong fear of infection among primary and secondary school students; 27% and 30%, respectively, and moderate to very high risk perception; 27% and 40%) (Table 2). Moreover, staff members with medium to very strong fear of infection were generally older and suffered more often from chronic conditions compared to those with no to a bit of fear (median (range) age, 49.0 (30.0, 65.0) vs. 44.0 (28.0, 65.0) years, and 55.7% (39/70) vs. 33.8% (26/77)).

### 3.6. Individual-Level Health Behaviours

Overall, recommended individual-level health behaviours were adhered to well (Table 2). Almost 70% of all participants reported washing their hands or using disinfecting hand-rub at least five times per day. Among school staff, 88% fell in this response range, compared to 65% of primary and 56% of secondary school students. Physical distancing at school and in public was followed frequently or always by over 70% of all participants. As with hand-rub use, this proportion was highest among staff, less among primary school students, and least in older students (Table 2). The highest proportion of frequent or continuous facemask use in school was found among primary school students (50%), followed by older students (35%) and school staff (40%). On the other hand, more than half (54%) of secondary school students reported wearing a facemask never or rarely at school. In staff and primary school students this was true for about 40%. The most popular type of facemask was one made of fabric or cloth (primary school students, 76.9%, 133/173; secondary school students, 63.1%, 113/179; staff, 78.6%, 103/131), followed by a surgical mask (32.4%, 56/173; 50.3%, 90/179; 39.7%, 52/131; on a multiple response scale).

### 3.7. School-Level Infection Prevention Measures

**Figure 2:**
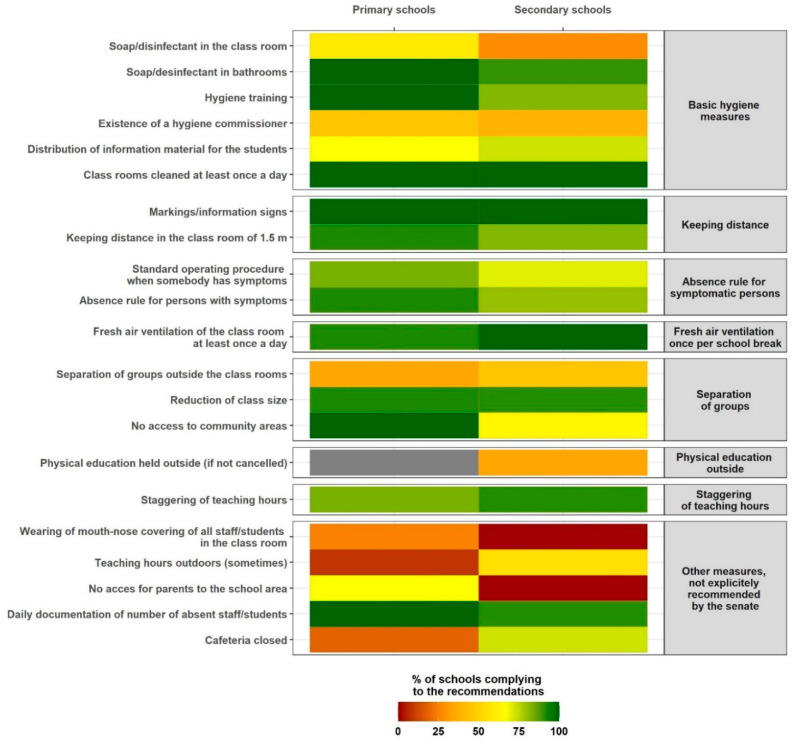
Heat map with percentage of implementation of the preventive measures in classes, stratified by the main aspects of the senate’s recommendations valid in June 2020.

Data on the implementation of obligatory IPC measures and recommendations in the visited schools are displayed in Figure 2. Highest adherence rates were observed for keeping distance and fresh air ventilation. Basic hygiene measures, such as daily cleaning of the classroom, were implemented at every school, but less than half of schools had a hygiene commissioner. While the majority of schools had reduced class sizes at the time of the study, class cohorting outside of the classroom was practiced in less than half of the facilities. In primary schools, sports activities were suspended, and only a minority of secondary school classes had physical education outside instead of inside. All schools implemented measures going beyond governmental requirements at that time, including wearing facemasks, teaching hours outdoors, restricted parental access, daily documentation of absent staff and students, and closure of the canteen. The implementation rate of these additional measures was very heterogeneous. Overall, more primary schools implemented preventive measures compared to secondary schools (Figure 2). As for distanced learning, 66.7% (14/21) of the classes reported some kind of online teaching. On average, 15% (range, 0–50%) of teaching was held online at primary schools and 50% (range, 0–90%) at secondary schools. In total, three classes (12.5%; all at primary schools) reported that all persons in the class were wearing masks.

## 4. Discussion

Among students and staff in Berlin schools during the early post-lockdown phase, the prevalence of SARS-CoV-2 infections (0.2%) and IgG sero-reactivity (1.3%) was low. Our study also shows that, even in summer, mild symptoms resembling a common cold are frequently reported at school (20%) and that the bulk of health impairment was not due to SARS-CoV-2 infection. Furthermore, the then-existing absence rule for symptomatic persons might have led to underreporting. The large proportion of affected children (even larger considering the preceding two weeks) poses a dilemma to parents, school staff, and policy-makers. In this context, the Berlin senate issued regulations that allowed regular school attendance for students with only mild and afebrile symptoms, e.g., rhinorrhoea, cough. Nevertheless, SARS-CoV-2 infection often runs an asymptomatic course in children and adolescents [24], and the mild symptoms of the one infected juvenile diagnosed in this study could have easily been overlooked. Vice versa, specific symptoms suggestive of SARS-CoV-2 infection, e.g., loss of smell and taste, were very rare. In the absence of routine testing of students, e.g., by antigen tests, asymptomatic SARS-CoV-2 infections in school presumably need to be tolerated. In this regard, low child-to-child transmission rates in educational settings are reassuring [11,17], but it remains to be seen whether this holds true at higher infection incidence, or with SARS-CoV-2 mutants emerging. In December 2020, Germany released antigen tests for lay-use by teachers and educators (but not by students or parents), which constitutes a good starting point considering that most individuals in school-based outbreaks so far have been adults [12]. Nevertheless, routine testing of students (taking into account asymptomatic infections), e.g., twice a week, is a desirable next step to prevent viral transmission within and outside of school. School staff reported a high prevalence of chronic conditions (44%), half of which were not specified. This may reflect the teachers’ relatively high age in the present study, as is the case across Germany [25]. Thus, a substantial fraction of teachers may belong to a COVID-19-relevant risk group. A 2015 review on German teachers’ health showed that half of a teachers’ cohort was overweight with 13% classed as obese, and 48% suffered from hypertension [26], factors that may contribute to more severe courses of COVID-19. Yet, the high reporting frequency of indeterminate chronic conditions might also point to a more general self-awareness of vulnerability among teachers. One-third of teachers reportedly experience exhaustion and high emotional workload [26], enforcing perceived vulnerability and susceptibility to health hazards. Accordingly, school staff in our study frequently reported fear of infection and self-classified as being at high risk. This perception finds reflection in the high adherence to IPC measures like hand hygiene and facemask-wearing. Policymakers should take into consideration that teachers and educators, while adapting to COVID-19-related changes, need clear instructions for a safe workspace to avoid psychological distress and unnecessary school absenteeism.

Previous studies have shown unfavourable changes in social life and physical activity due to school closures [27,28]. Our results show that much less time was spent meeting friends compared to pre-pandemic times among both students and staff. However, our instruments were not designed for an in-depth assessment of the extent of social isolation and socio-affective conditions potentially arising from that, as reported elsewhere [3,29]. Self-reported screen time increased particularly among students. Physical activity dropped among primary school pupils, but much less among older students. Data obtained during the COVID-19 pandemic in China with its very strict lockdown showed a sharp increase in screen time, in parallel with a significant decrease in physical activity [27]. While those results are transferable to the Berlin context only to a limited extent, our findings confirm this tendency especially among younger children and give rise to concern: excessive screen time in conjunction with sedentary behaviour, snacking, and weight gain [28] have been associated with cardiovascular disease risk factors such as obesity, high blood pressure, and insulin resistance [6]. Depending on the duration of the pandemic, there is a risk that some of the newly established behaviours may persist, requiring parents, teachers, and policy-makers to promote healthy lifestyles.

Our findings show that the implementation of IPC measures in schools is feasible, as the governmental recommendations were largely implemented, with primary schools performing better than secondary schools. This might partially reflect more dismissive attitudes towards regulations, especially on social behaviour, among older students or more flexible adaption in primary schools, which tend to be smaller. The latest official recommendations on school operation of November 2020 strengthen fresh air ventilation and hand hygiene, and present separate advice for primary and secondary schools in the form of a four-tier system to enable adaptation to the local situation [22]. Nevertheless, preventive measures at the school and class level will continuously need to be adapted, mirroring and anticipating relevant epidemic developments.

Our study has several limitations. Sample size and study period pose limitations to the generalizability of our data. Voluntary participation of both institutions and school members and low participation rates in some facilities may have caused a selection bias, limiting generalizability. Further data from other settings are thus required for the confirmation of our findings. Determinants of infection, including association between adherence to IPC measures and transmission, could not be assessed due to the detection of a single case only. On the other hand, sample collection among children and adolescents was unproblematic as reflected by the high proportion of available specimens. Our findings suggest that educational settings and their players are largely able to adapt to IPC measures and to changing conditions. Increased screen time as well as reduced social contacts and, partially, physical activity point to non-infectious dangers brought on by the pandemic. The needs and situational requirements of students and teachers are to be met, including those linked to fear and behaviour. These form the prerequisites for the comprehension of and adherence to IPC measures, which in turn determine school functioning. Ongoing follow-up examinations will show whether this can be achieved. In the meantime, regular screening of students and teachers for SARS-CoV-2 in the school setting may help to reduce both infections and uncertainties, thereby ensuring the right to education.

## Figures and Tables

**Table 1:** Reported symptoms on examination day, during the preceding 14 days, and medical history.

	Primary School Students (*N* = 193)		Secondary School Students (*N* = 192)		Staff (*N* = 150)	
	%	*n/N*	%	*n/N*	%	*n/N*
On examination day:						
Any of the defined symptoms	18.8	36/192	16.3	31/190	12.2	18/148
Headache	6.8	13/192	6.8	13/192	7.4	11/148
Rhinorrhoea	7.8	15/192	7.8	15/192	2.0	3/148
Cough	5.7	11/192	2.6	5/192	3.4	5/148
Sore throat	3.1	6/192	3.6	7/192	0.7	1/148
Diarrhoea	2.1	4/192	0	0/192	1.4	2/148
Limb pain	1.0	2/192	0	0/192	0	0/148
Loss of smell and taste	0.5	1/190	0.5	1/190	0	0/148
Self-reported fever	1.6	3/192	0	0/192	2.0	3/148
Fever ≥ 37.5 °C; measured	2.1	4/192	3.1	6/192	1.3	2/149
During preceding 14 days:						
Any of the defined symptoms	40.6	76/187	54.9	101/184	48.0	71/148
Headache	21.5	41/191	35.4	67/189	32.9	49/149
Rhinorrhoea	11.1	21/190	20.2	38/188	7.5	11/146
Cough	5.3	10/190	11.6	22/189	4.7	7/149
Sore throat	7.0	13/187	14.4	27/187	6.9	10/145
Diarrhoea	7.0	13/186	4.9	9/184	7.6	11/145
Limb pain	3.7	7/189	5.4	10/186	3.5	5/144
Loss of smell and taste	0	0/191	1.6	3/185	0	0/147
Breathlessness	4.7	9/192	11.1	21/189	6.1	9/148
Tiredness	4.8	9/188	8.0	15/188	18.8	28/149
Chills	0	0/187	1.6	3/187	2.1	3/146
Chest pain	1.6	3/188	4.3	8/187	3.4	5/146
Self-reported fever	0.5	1/190	0.5	1/189	2.0	3/149
Self-reported chronic diseases						
Any	13.0	25/192	18.1	34/188	44.2	65/147
High blood pressure	0	0/193	0	0/192	14.0	21/150
Chronic heart disease	0	0/193	2.1	4/192	4.7	7/150
Obesity	1.0	2/193	0	0/192	6.7	10/150
Diabetes mellitus	0	0/193	0	0/192	0.7	1/150
Chronic lung disease	4.1	8/193	4.2	8/192	4.0	6/150
Immunodeficiency	0.5	1/193	0.5	1/192	1.3	2/150
Cancer	0	0/193	0	0/192	2.0	3/150
Other	8.8	17/193	10.4	20/192	21.3	32/150
Regular medication	7.8	15/192	12.2	23/188	39.3	57/145
Allergies	29.4	55/187	26.5	48/181	40.7	57/140
Smoking	-	-	5.3	10/188	23.8	35/147

**Table 2:** Proportions for reported fear of infection, risk perception, and health behaviours.

	Primary School Students (*N* = 193)		Secondary School Students (*N* = 192)		Staff (*N* = 150)	
	%	*n/N*	%	*n/N*	%	*n/N*
Fear of infection						
Not at all to a bit	72.9	137/188	70.0	133/190	51.7	77/149
Medium to very strong	27.1	51/188	30.0	57/190	48.3	72/149
Risk perception						
Very low to rather low	72.9	137/188	60.5	115/190	40.9	61/149
Moderate to very high	27.1	51/188	39.5	75/190	59.1	88/149
Hand washing/using handrub per day						
0–1 time	1.0	2/192	4.7	9/191	0	0/149
2–4 times	34.4	66/192	39.3	75/191	12.1	18/149
≥5 times	64.6	124/192	56.0	107/191	87.9	131/149
Physical distancing at school						
Never to rarely	2.6	5/189	15.8	30/190	3.4	5/147
Sometimes	16.4	31/189	29.5	56/190	19.7	29/147
Frequently to always	81.0	153/189	54.7	104/190	76.9	113/147
Facemask wearing at school						
Never to rarely	38.9	74/190	53.7	102/190	38.5	57/148
Sometimes	11.1	21/190	11.1	21/190	21.6	32/148
Frequently to always	50.0	95/190	35.3	67/190	39.9	59/148
Situations of facemask wearing in the school (multiple response scale)						
Never	32.9	55/167	29.4	55/187	25.5	36/141
In class	38.3	64/167	9.6	18/187	54.6	77/141
In schoolyard	32.9	55/167	35.8	67/187	22.7	32/141
On way to school	24.0	40/167	48.1	90/187	24.8	35/141

## Data Availability

The data presented in this study are available on request from the corresponding author.
